# Assessing the Work Activities Related to Musculoskeletal Disorder among Critical Care Nurses

**DOI:** 10.1155/2021/8896806

**Published:** 2021-06-29

**Authors:** Aesha Abdullah Aleid, Hend Abdelmonem Eid Elshnawie, Ahmed Ammar

**Affiliations:** Imam Abdulrhman Bin Faisal University, Dammam, Saudi Arabia

## Abstract

Nurses are physically exhausted with an incidence of work-related musculoskeletal disorders (MSDs), especially those working in critical care units that require exhaustive physical efforts to fulfill patients' needs. The current study aims to assess work activities related to the occurrence of MSDs in nurses working in critical care units. A descriptive cross-sectional study was conducted on a sample of 100 nurses of critical care units, and the data were collected via a survey questionnaire. The study was conducted at King Fahad University Hospital for three months from February to April 2019. The study findings revealed that work activities related to MSD were associated with sociodemographic data, age, and neck pain (rho = 0.063) (*p*=0.03) and lower back pain (rho = 0.89) (*p*=0.03); education level with hip/thigh pain (rho = 0.64) (*p*=0.03); standing time with shoulder pain (rho = 0.66) (*p*=0.04), wrist/hand pain (rho = −0.75) (*p*=0.05), hip/thigh pain (rho = −0.78) (*p*=0.004), and knee pain (rho = −0.77) (*p*=0.005). An increased prevalence of MSDs with lower back pain (92%) and upper back pain (56%) was estimated among the nurses, and a negative impact of work-related MSDs on occupational health and daily life activities of the nurses was also observed. The study concluded that the occurrence of MSDs is significantly associated with sociodemographic data: age, BMI experience level, and educational level of nurses of critical care units.

## 1. Introduction

Musculoskeletal disorder (MSD) is a condition associated with damage to the body tissues caused by continuous physical strain and trauma. This trauma causes pain and discomfort because of various inflammatory symptoms that affect muscles, tendons, ligaments, joints, blood vessels, and peripheral nerves [1, 2]. There is an increased prevalence of MSDs among the nurses of critical care units which significantly affects their health [3]. According to Bernal et al. [4], long-term exposure to a specific task at the workplace causes pain in muscles, bones, tendons, blood vessels, and nerves which eventually leads to MSDs.

Nursing is considered as one of the most exhausting jobs that are associated with an increased incidence of work-related musculoskeletal disorders (MSDs) [5]. Nurses are reported to experience an increased prevalence of MSDs as compared to other system disorders [6]. According to the recent statistics [7], there is an increase in the prevalence of MSDs across developing as well as developed countries. As compared to other clinical settings, the nurses working in critical care units exert much physical effort to fulfill patient needs, which increases their susceptibility to MSDs [8, 9]. A recent study conducted by Amer [5] reported that nurses working in Intensive Care Unit are at increased risk of developing lower back pain (79.3%), knee pain (72.4%), shoulder pain (72.4%), neck pain (62.1%), and elbow pain (8.6%).

One of the major risk factors leading to the development of musculoskeletal disorders is work activities. Work activities that cause extreme fatigue in the lower and upper extremities, lower back, and neck lead to MSDs and such diseases are termed as occupational diseases [10]. One of the previous studies by Purani et al. [11] reported that MSDs develop because of the constant repetition of improper postures and movements during work. Another recent study by Epstein et al. [12] has affirmed that the risk of developing MSDs increases due to force of movement, monotonous tasks, lack of control over job, and poor communication skills. Besides, work conditions like pace of work, weight of handling object, and layout of working station also significantly influence the development of MSDs [13]. The development of musculoskeletal pain was discussed by Lietz et al. [14] and the analysis showed that the risk of MSDs increases on account of the use of vibrating equipment, adoption of consistent static posture, and utilization of psychomotor skills. Therefore, it can be affirmed that physical factors along with the occupational setting are correlated in the development of MSDs.

The occurrence of MSDs is generally higher among critical care nurses than that of nonspecialized nurses. This is specifically accurate for scrub nurses who are more prone to MSDs as being actively engaged to create and maintain the surgical field and passing medical equipment to surgeons [15]. Idiosyncrasies of their work tasks includes unusual motions, implementation of awkward and static postures, and continuous repetitive movements for protracted time periods, and lifting and holding up immense surgical instruments when aiding the surgeon and caring for the patient. [16]. In particular, critical care nurses have an even higher risk to develop MSDs if working full-time as critical nurses.

A lot of efforts and skills are required from the nurses of critical care units for catering their patient's need and, therefore, the risk of development of MSDs in them increases accordingly. It is a well-known fact that if a body position is maintained for a long time, it can lead to discomfort and fatigue [17]. Similarly, nurses of the critical care units hold their neck and shoulders in a fixed position and this consistent posture leads to the development of MSDs characterized by injuries and pain. For the last two decades, the development of MSDs has been reported more frequently in the nurses of critical care units as compared to the nurses of clinical care units. The development of MSDs in the nurses of the critical care units is a major setback for them as not only their treatment options are expensive but also do they reduce the quality of patient care. There is a need to increase awareness in the nurses of critical care units regarding the usage of proper procedures of handling and movements of patients/objects via developing coping strategies, educational programs, and implementation of special policies and procedures.

Previous studies have shown that the adoption of effective coping strategies and educational programs minimizes the incidence of work-related MSDs in the nurses and also increases their efficiency in giving quality care to the patients [18, 19]. These studies have successfully identified several work-related MSDs and their treatment costs. However, none of the previous studies provided relevant information for the policymakers and planners to plan adequate policies and procedures to reduce staff absenteeism and in turn increase the quality of work with positive outcomes.

The role of occupational therapy entails adaptation and changes in work tasks, prevention of injury via education of employees and management, and ergonomic assessments. A critical care nurse would undertake the vocational element of the nurse with MSDs and assess the factors contributing to the injury throughout the work environment [20]. Research by Abu Tariah et al. [21] offers evidence for the effectiveness of interventions for preventing MSDs. An ergonomic assessment by the OT comprises the following movements to investigate the physical stress that it causes and mitigate adverse postures or repetitive muscle use. Changes to the task are suggested for enhancing body mechanics. The occupational therapists would further contribute to modifying the engineering controls of any transportation necessities of work tasks [22].

Therefore, the present study aims to determine work activities that lead to the development of MSDs in the nurses of critical care units and to provide relevant information required to devise preventive measures to manage those activities properly in the workplace. The study results would help in developing better programs and procedures for preparing nurses of critical care units by providing them with adequate knowledge about how to minimize the risk factors of MSDs. The research questions addressed by the study are as follows:

- What are the major risk factors that lead to the development of MSDs in nurses of critical care units?
- Is there a significant association between demographic characteristics and occurrence of MSDs in nurses of critical care units?

## 2. Subjects and Methods

### 2.1. Setting

This study employed a cross-sectional study design to assess the work activities related to MSDs in nurses of critical care unit working at King Fahad University Hospital located in the city of Al-Khobar in the eastern province of Saudi Arabia. The study was conducted in the critical care unit of King Fahad Hospital for three months between February and April 2019.

### 2.2. Ethical Approval

This study approval was obtained from the Institute Review Board (IRB) of Imam Abdulrahman Bin Faisal University and the permission of the director of research of King Fahad Hospital was also acquired. Additionally, informed consent was duly obtained from each participant before recruiting them for the study.

### 2.3. Participants

**Figure 1:**
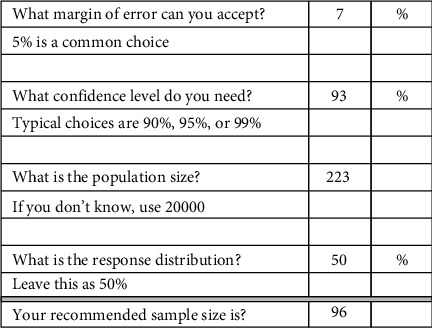
Sampling criteria.

A convenience sample of all nurses working in the surgical adult intensive care unit, medical adult intensive care unit, cardiac care unit, and emergency department were surveyed with a confidence level of 93% and a margin error of 7%. The final sample included 100 nurses from critical care units who were exposed to the risk of developing MSDs because of consistent use of high and complex technologies. Following were the inclusion criteria for the study sample: the registered nurses who were working full-time in the emergency department and intensive care unit and directly involved in patient care. The exclusion criteria included part-time nurses, pregnant nurses, and those with recently diagnosed MSDs. In the first place, the voluntariness was ensured by asking participants to voluntarily participate in the study and the nurses who were not willing to participate in the study voluntarily were excluded. A total of 223 nurses were approached for data collection; however, only 100 questionnaires were received with complete details, with a response rate of 44.8% (Figure 1).

### 2.4. Data Collection

A structured close-ended questionnaire was used as a data collection instrument that comprised two parts:

- Part 1 gathered information related to gender, age, education level, height/weight, place of work, experience level, health problems, and awareness level through educational programs.
- Part 2 included a self-administered questionnaire containing 29 items that investigated the effect of work activities on the development of MSDs.

This prevalidated questionnaire was adapted from Bin Homaid et al. [23]. Approval was obtained from the author through e-mail before adopting the questionnaire. The tool used for data collection consisted of 29 items (closed-ended structured questions) that were grouped into the following two domains:

- Risk factor of MSD (15 questions): it contained questions related to stress level at work (1 question), smoking (1 question), period of long standing and setting (2 questions), and weight and type of lifting objects and number of caring (object or patient) (3 questions), body mechanism for lifting or performing procedure (3 questions), and transfer and moving patient (5 questions).
- MSD assessment in the nurses of critical care units (14 questions): it consisted of questions related to the location of aching area (1 question), nature of pain (5 questions), severity of pain (1 question), medical advice and treatment (4 questions), and effect and management of MSD-associated pain (3 questions).

The severity of pain was measured according to the Likert scale from 0 to 5: ranging from no pain to severe pain.

### 2.5. Data Analysis

The data collected through the questionnaire were entered, coded, and analyzed with the help of the IBM SPSS Statistic Program version 25.0. Descriptive statistics in the form of frequencies/percentages were used to present the categorical data, whereas mean/standard deviations were used to present the continuous data. Spearman's rank correlation coefficient was used to measure the association between sociodemographic factors and work-related activities to musculoskeletal dysfunction. *p*-value of less than 0.05 was considered statistically significant.

## 3. Results

The sociodemographic characteristics of nurses, including gender, age, BMI, education level, work experience in critical care unit, prolonged standing shift, and prolonged sitting shift, are presented in Table 1. The distribution of work activities leading to MSDs in the nurses of the critical care unit and the prevalence of musculoskeletal disorders as responded by the nurses are demonstrated in Table 2.

Table 3 shows a statistically significant association of ankle/feet pain with normal weight (58%), overweight (37%), and obesity (2%) (*p*-value = 0.02). However, none of the other MSDs showed a statistically significant association with BMI. The association of 2-year work experience at critical care unit with MSDs in the nurses is presented in Table 4.

The association of sociodemographic characteristics with MSDs in the nurses of the critical care unit is presented in Table 5. The results show a significant association of age with lower back pain (*p* value=0.03) and neck pain *p* value=0.03); education level with thigh/hip pain (*p* value=0.03); standing time with shoulder pain (*p* value=0.04), knee pain (*p* value=0.05), thigh/hip pain (*p* value=0.04), and hand/wrist pain (*p* value=0.04).

Table 6 shows the association between the work activities and MSDs related to upper and lower body. The results clearly depict that there is a statistically significant and positive correlation in bending to lift item from floor with neck pain (*p*=0.04) and upper back pain (*p*=0.03), lower back pain (*p*=0.004), hip/thigh pain (*p*=0.05), and knee pain (0.04).

## 4. Discussion

The results of the current study have supported the universal hypothesis which states that work activities extensively expose the nurses of critical care units to MSDs. Similar results were deduced by Amer [5] and the results showed a significant relationship between work activities and MSDs in the nurses of critical care units. The increased prevalence of MSDs in the nurses is related to various risk factors that include long working shifts, working in awkward positions, stressful physical working conditions, and manual handling of patients. The prevalence of MSDs also increases with progressing age as 49% of the affected nurses were aged between 25 and 30 years and 39% were aged between 31 and 35 years. The present study also shows a statistically significant relation of developing MSDs with lower back pain and neck pain (*p* value=0.03). These findings are supported by Tinubu et al. [2].

Many similar studies stated that nurses aged between 31 and 40 years were highly prone to develop MSDs. Attar [24] found that MSDs were highly prevalent in nurses aged more than 30 years unlike the nurses aged less than 30 years. This variation may also be due to the difference in tasks and procedures assigned to nurses in different clinical settings. The nurses of critical care units with experience of 2 years were likely to develop knee pain (*p* value=0.000), shoulder pain (*p* value=0.000), and thigh/hip pain (*p* value=0.01). The present findings show that most of the nurses (44%) experienced initial symptoms of MSDs in the first two years of their clinical practice and this result was in line with the results of Tinubu et al. [2].

There is a significant positive association of BMI with the development of MSDs in the form of feet and ankle pain (*p* value=0.02). These findings are in agreement with the study conducted by Aljerian et al. [25] reporting lower back pain, knee pain, and upper back pain among 63.3%, 41.4%, and 40% of the nurses of critical care units. On the contrary, Bin Homaid et al. [24] identified no significant association between MSDs and demographic characteristics of nurses including their age, BMI, and working conditions. A recent study conducted by Yan et al. [26] also found no association between work experience (6–15 years) and development of MSDs in the nursing staff. This inconsistency might be attributed to the age of the nurses considered in both studies.

According to the current study, a long-standing time of 5 to 8 hours is statistically significant for developing MSDs in the form of knee pain (*p* value=0.005), hand/wrist pain (*p* value=0.004), and thigh/hip pain (*p* value=0.005) among the nurses of critical care units. In a similar context, Bernal et al. [4] identified that prolonged standing increased the risk of spinal loading and shrinkage. It has also been observed that, as compared to the more educated nurses, less educated nurses were at greater risk of experiencing symptoms of MSDs [27]. This might be because more educated nurses are well aware of the policies and procedures of their tasks.

Concerning the MSDs, the current study reported that most of the nurses (92%) suffered from lower back pain, which was followed by upper back pain (56%), shoulder pain (36%); neck pain (31%), ankle pain (25%), hand/wrist pain (21%), knee pain (20%), thigh/hip pain (19%), and elbow pain (14%). The high rate of lower back pain was also reported by Amer [5] as his study showed that most of the nurses (79.3%) suffered from lower back pain. These results are further corroborated by the study of Aljerian et al. [28] which showed that more than three-quarters of the nurses suffered from lower back pain followed by neck pain. On the contrary, few studies narrated that the lower back was the least affected body part in the nurses [27, 29]. This variance may be attributed to various procedures, activities, or tasks performed by the nurses.

In addition, critical care nurses must retain in critical situations at all times, affecting awkward and prolonged static postures, upper extremity strain, and constant trunk flexion. Therefore, a prolonged static position is ideally recognized to be more disadvantageous as compared to a dynamic posture because of higher levels of lactic toxins and acidosis caused by the former.

Some of the previous studies reported that neck bending and pushing/pulling heavy objects were the most perceived job risk factors [28]. Similarly, the current study also highlights carrying, lifting, or moving heavy material or equipment as the major risk factors leading to MSDs. The differences found in the prevalence of work-related risk factors are attributed to the differences in the working conditions and tasks performed by the nurses in different units, hospitals, and countries. In fact, surgical instruments are rarely designed based on standard ergonomic principles that would ease their handling by health care providers. The ergonomic circumstances can be an essential factor in addition to the ergonomic design of surgical instruments. Surgical instrument makers must undertake encompassing professional employees into the design of surgical instruments for encompassing user's experience. In addition, healthcare professionals must be involved in the design and remodeling of the operating room for creating a user-centered work environment. Additionally, adversely designed instruments can indeed elevate hand, finger, and wrist strains of operators, which make these subjects more prone to developing upper limb disabilities. Therefore, a more operator-friendly design would definitely assist in reducing the physical strain for critical care nurses as well as surgeons in the operating room.

It is quite clear that an appropriate assessment of the physical strain endured by critical care nurses would benefit the integration of occupational health measures intended to reduce the risk of MSDs. Therefore, it is important for introducing innovative technical measurements able to assess objectively the physical workload for determining particular ergonomic risk factors. A task analysis is assumed a more viable approach for conducting this type of evaluation since motion sensors and electrodes could interfere with activities of operators. In addition, a task analysis would facilitate in order to assess type-specific surgical procedures on the basis of a holistic approach undertaking all most appropriate components of human skills.

Even though the current study is helpful for the development of specific programs and policies for the nurses of critical care nurses, there are also some limitations. The small sample size considered in this study could not be used for obtaining generalized findings across different regions in Saudi Arabia. Moreover, some of the collected data including information about prevention method and specific treatment were subjective; therefore, it may provide exaggeration or be presented as a limiting factor. Another limitation of our study is that albeit the response rate was good, a nonresponse analysis could not be performed due to the lack of nonrespondent data. Consequently, a bias due to selective nonresponse cannot be ruled out. For these reasons, our results should be interpreted with caution. Finally, the study results are not completely justified with literature support as there are only a few studies that have focused on the assessment of the quality of nurses with respect to MSDs.

## 5. Conclusion

The results of this study demonstrate a significant association between sociodemographic characteristics (age, BMI, and work experience) and development of MSDs in nurses of critical care units. The work activities having a significant association with the development of MSDs include repositioning of patients in bed, bending to lift items from floor, and lifting object above waist. Moreover, clinical characteristics such as prolonged standing time and increased stress level have been found to be significantly associated with MSDs. The results show that the majority of the nurses suffered from lower back pain (92%) and upper back pain (56%). There was a significant negative impact of the stated work activities on the quality of patient care provided by the nurses of critical care units. Based on the results deduced in this study, it is recommended that critical care unit nurses need to be educated about the risk factors that may lead to the development of MSDs and effective strategies should be formulated for utilizing proper procedures to move, handle, and position the patients of critical care unit. Concerning the clinical practice, the study suggests that the critical care unit should be equipped with an adequate number of nurses to minimize work pressure on each nurse in order to avoid the development of MSDs. Moreover, an adequate number of nurses should be hired in critical care setting of nurses to avoid MSD development. However, future studies need to focus on the variables that initiate MSDs including stress, resilience, work environment, fatigue, and sleep privation. Examination of the work activities that lead to MSDs in nurses of different regions should be thoroughly conducted to promote the generalization of study findings.

## Figures and Tables

**Table 1:** Demographic profile of the nurses.

Item	Measure	N (%)
Gender	Male	23 (23%)
	Female	77 (77%)

Age	25–30 years	49 (49%)
	31–35 years	39 (39%)
	36–40 years	5 (5%)
	41–55 years	3 (3%)
	>56 years	1 (1%)

BMI	<18.5 (underweight)	2 (2%)
	18.5–24.9 (normal)	58 (58%)
	25–29.9 (overweight)	38 (38%)
	>30 (obese)	2 (2%)

Education level	Nursing diploma	10 (10%)
	Nursing bachelor	86 (86%)
	Postgraduate	4 (4%)

Work experience	1–5 years	32 (32%)
	6–10 years	48 (48%)
	>10 years	20 (20%)

Critical care area experience	<1 year	16 (16%)
	1–2 years	40 (40%)
	>2 years	44 (44%)

Prolonged standing time during shift	1–4 hours	20 (20%)
	5–8 hours	60 (60%)
	>8 hours	20 (20%)

Prolonged sitting time during shift	1–4 hours	88 (88%)
	5–8 hours	7 (7%)
	>8 hours	5 (5%)

**Table 2:** Work activities leading to MSDs and prevalence of musculoskeletal disorders.

Item	N (%)
*Work activities leading to MSDs*	
Patient lifting (bed ridden)	(*n* = 92) 92%
Repositioning patient in bed	(*n* = 91) 91%
Pulling patient up the bed	(*n* = 90) 90%
Transfer patient onto a stretcher	(*n* = 83) 83%
Ambulating a patient	(*n* = 82) 82%
Bending to lift item from floor	(*n* = 78) 78%
Transfer patient to bed/chair	(*n* = 77) 77%
Lifting objects above the waist	(*n* = 56) 65%
Rotating torso while bearing weight	(*n* = 57) 57%

*Prevalence of musculoskeletal disorders*	
Lower back pain	(*n* = 92) 92%
Upper back pain	(*n* = 56) 56%
Shoulder pain	(*n* = 36) 36%
Neck pain	(*n* = 31) 31%
Ankle/feet pain	(*n* = 25) 25%
Wrist/hand pain	(*n* = 21) 21%
Knee pain	(*n* = 20) 20%
Hip/thigh pain	(*n* = 19) 19%
Elbow pain	(*n* = 14) 14%

**Table 3:** Association of BMI related to MSDs.

MSDs *n* (100%)	Underweight 3 (3%)	Normal weight 58 (58%)	Overweight 37 (37%)	Obese 2 (2%)	*p* ^*∗*^-value
Neck pain	3.2	61	32	3.2%	0.35
Shoulder pain	2	47.2	47.2	2.8	0.46
Elbow pain	0	64.3	35.7	0%	0.58
Wrist/hand pain	0	11	10	0%	0.76
Upper back pain	1.8	60.7	33.9	3.6%	0.35
Lower back pain	2.2	56.5	38	3.3%	0.37
Hip/thigh pain	0	57.9	42.1	0%	0.88
Knee pain	0	45	55	0%	0.36
Ankle/feet pain	0	36	60	4%	0.02^*∗*^

^*∗*^Chi-square.

**Table 4:** Association of 2-year work experience at critical care unit with MSDs.

*p* ^*∗*^-value	Correlation coefficient	MSDs (100%) (*n* = 100)	Critical care area experience year
0.910	0.011	Neck pain	>2 years
0.001^∗^	−0.318	Shoulder pain	
0.070	−0.182	Elbow pain	
0.370	−0.091	Wrist/hand pain	
0.753	0.032	Upper back pain	
0.848	−0.019	Lower back pain	
0.010^∗^	−0.255	Hip/thigh pain	
0.000^∗^	−0.343	Knee pain	
0.116	−0.158	Ankle/feet pain	

^*∗*^Spearman test.

**Table 5:** Association of sociodemographic related to MSDs among critical care nurses.

*p* ^*∗*^-value	Correlation coefficient	MSDs	Sociodemographic
0.03^*∗*^	0.63	Neck pain	Age
0.91	0.12		Education level
0.73	0.03		Standing time
0.67	0.04		Sitting time

0.14	−0.15	Shoulder pain	Age
0.92	−0.01		Education level
0.04^*∗*^	0.66		Standing time
0.43	0.07		Sitting time

0.67	−0.04	Elbow pain	Age
0.40	−0.10		Education level
0.07	−0.18		Standing time
0.21	−0.12		Sitting time

0.39	−0.08	Wrist/hand pain	Age
0.47	−0.08		Education level
0.05^*∗*^	−0.75		Standing time
0.67	−0.04		Sitting time

0.17	0.13	Upper back pain	Age
0.61	−0.07		Education level
0.75	−0.03		Standing time
0.08	0.17		Sitting time

0.03^*∗*^	0.89	Lower back pain	Age
0.94	−0.05		Education level
0.56	−0.05		Standing time
0.99	−0.00		Sitting time

0.78	−0.02	Hip/thigh pain	Age
0.03^*∗*^	−0.64		Education level
0.004^*∗*^	−0.78		Standing time
0.53	0.06		Sitting time

0.08	−0.17	Knee pain	Age
0.41	−0.08		Education level
0.005^*∗*^	−0.77		Standing time
0.53	−0.06		Sitting time

0.26	−0.11	Ankle/feet pain	Age
0.34	−0.09		Education level
0.14	−0.14		Standing time
0.49	0.07		Sitting time

^*∗*^Spearman test.

**Table 6:** Association of work activities related to upper and lower body MSDs among studied critical care nurses.

*p* ^*∗*^-value	Correlation coefficient	MSDs	Work activities
*Upper body MSDs*			
0.15	0.14	Neck pain	Heaviest object lifted
0.20	0.12		Lifting objects above the waist
0.88	0.01		Rotating torso while bearing weight
0.04^*∗*^	0.99		Bending to lift item from floor
0.56	0.05		Transfer patient to bed/chair
0.06	0.18		Transfer patient onto a stretcher
0.37	0.08		Ambulating a patient
0.13	0.15		Pulling patient up the bed
0.18	0.13		Repositioning patient in bed

0.13	0.15	Shoulder pain	Heaviest object lifted
0.79	0.02		Lifting objects above the waist
0.29	−0.10		Rotating torso while bearing weight
0.14	0.14		Bending to lift item from floor
0.26	0.11		Transfer patient to bed/chair
0.94	0.00		Transfer patient onto a stretcher
0.79	0.02		Ambulating a patient
0.27	0.11		Pulling patient up the bed
0.10	0.13		Repositioning patient in bed

0.42	−0.081	Elbow pain	Heaviest object lifted
0.51	−0.09		Lifting objects above the waist
0.08	−0.17		Rotating torso while bearing weight
0.95	−0.00		Bending to lift item from floor
0.88	0.01		Transfer patient to bed/chair
0.21	−0.12		Transfer patient onto a stretcher
0.25	0.11		Ambulating a patient
0.56	−0.05		Pulling patient up the bed
0.20	0.12		Repositioning patient in bed

0.84	−0.020	Wrist/hand pain	Heaviest object lifted
0.74	−0.03		Lifting objects above the waist
0.33	−0.09		Rotating torso while bearing weight
0.12	0.15		Bending to lift item from floor
0.29	0.10		Transfer patient to bed/chair
0.35	−0.09		Transfer patient onto a stretcher
0.89	−0.01		Ambulating a patient pulling patient up the bed
0.93	0.00		
0.45	0.07		Repositioning patient in bed

0.52	−0.64	Upper back pain	Heaviest object lifted
0.27	0.11		Lifting objects above the waist
0.66	0.04		Rotating torso while bearing weight
0.03^*∗*^	0.64		Bending to lift item from floor
0.37	0.90		Transfer patient to bed/chair
0.78	0.02		Transfer patient onto a stretcher
0.57	0.05		Ambulating a patient
0.28	0.10		Pulling patient up the bed
0.15	0.14		Repositioning patient in bed

*Lower body MSDs*			
0.209	0.12	Lower back pain	Heaviest object lifted
0.091	0.71		Lifting objects above the waist
0.25	0.11		Rotating torso while bearing weight
0.004^*∗*^	0.75		Bending to lift item from floor
0.31	0.10		Transfer patient to bed/chair
0.53	0.063		Transfer patient onto a stretcher
0.014^*∗*^	0.64		Ambulating a patient
0.14	0.14		Pulling patient up the bed
0.003	0.69		Repositioning patient in bed

0.38	0.08	Hip/thigh pain	Heaviest object lifted
0.16	0.14		Lifting objects above the waist
0.26	0.11		Rotating torso while bearing weight
0.05^*∗*^	0.67		Bending to lift item from floor
0.82	0.02		Transfer patient to bed/chair
0.87	0.16		Transfer patient onto a stretcher
0.78	0.02		Ambulating a patient
0.45	0.07		Pulling patient up the bed
0.13	0.15		Repositioning patient in bed

0.57	−0.056	Knee pain	Heaviest object lifted
0.60	0.052		Lifting objects above the waist
0.84	−0.02		Rotating torso while bearing weight
0.04^*∗*^	0.62		Bending to lift item from floor
0.34	0.95		Transfer patient to bed/chair
0.79	0.02		Transfer patient onto a stretcher
0.30	0.10		Ambulating a patient
0.09	0.16		Pulling patient up the bed
0.11	0.15		Repositioning patient in bed

1.00	0.17	Ankle/feet pain	Heaviest object lifted
0.02^*∗*^	0.73		Lifting objects above the waist
0.73	0.03		Rotating torso while bearing weight
0.40	0.08		Bending to lift item from floor
0.13	0.15		Transfer patient to bed/chair
0.17	0.13		Transfer patient onto a stretcher
0.76	0.03		Ambulating a patient
0.25	0.11		Pulling patient up the bed
0.07	0.18		Repositioning patient in bed

^*∗*^Spearman test.

## Data Availability

The datasets used and analyzed during the current study are available from the corresponding author upon reasonable request.
